# Performance-Enhancing Materials in Medical Gloves

**DOI:** 10.3390/jfb14070349

**Published:** 2023-06-30

**Authors:** María José Lovato, Luis J. del Valle, Jordi Puiggalí, Lourdes Franco

**Affiliations:** 1 Departament d’Enginyeria Química, Escola d’Enginyeria de Barcelona Est-EEBE, Universitat Politècnica de Catalunya, c/Eduard Maristany 10-14, 08019 Barcelona, Spain; maria.jose.lovato@upc.edu (M.J.L.); luis.javier.del.valle@upc.edu (L.J.d.V.); jordi.puiggali@upc.edu (J.P.); 2 Center for Research in Nano-Engineering, Universitat Politècnica de Catalunya, Campus Sud, Edifici C’, c/Pasqual i Vila s/n, 08028 Barcelona, Spain

**Keywords:** medical gloves, natural rubber, synthetic rubber, bio-filler, reinforcing filler, antimicrobial properties, performance-enhancing materials

## Abstract

Medical gloves, along with masks and gowns, serve as the initial line of defense against potentially infectious microorganisms and hazardous substances in the health sector. During the COVID-19 pandemic, medical gloves played a significant role, as they were widely utilized throughout society in daily activities as a preventive measure. These products demonstrated their value as important personal protection equipment (PPE) and reaffirmed their relevance as infection prevention tools. This review describes the evolution of medical gloves since the discovery of vulcanization by Charles Goodyear in 1839, which fostered the development of this industry. Regarding the current market, a comparison of the main properties, benefits, and drawbacks of the most widespread types of sanitary gloves is presented. The most common gloves are produced from natural rubber (NR), polyisoprene (IR), acrylonitrile butadiene rubber (NBR), polychloroprene (CR), polyethylene (PE), and poly(vinyl chloride) (PVC). Furthermore, the environmental impacts of the conventional natural rubber glove manufacturing process and mitigation strategies, such as bioremediation and rubber recycling, are addressed. In order to create new medical gloves with improved properties, several biopolymers (e.g., poly(vinyl alcohol) and starch) and additives such as biodegradable fillers (e.g., cellulose and chitin), reinforcing fillers (e.g., silica and cellulose nanocrystals), and antimicrobial agents (e.g., biguanides and quaternary ammonium salts) have been evaluated. This paper covers these performance-enhancing materials and describes different innovative prototypes of gloves and coatings designed with them.

## 1. Introduction

To minimize the risk of exposure to cross-infection between patients and healthcare workers, it is necessary to use personal protective equipment (PPE) such as disposable medical gloves, masks, or gowns [1]. Among these items, medical gloves were widely used by the population during the COVID-19 pandemic and played a key role as an infection prevention tool for medical staff and society in general. Microorganisms, infectious agents, and pathogens, such as bacteria, viruses, fungi, protozoa, and prions, live in the human body and the surrounding environment [2]. Most of these organisms do not pose a threat to the general population, but during an epidemic or in medical facilities, pathogenic microorganisms can be present at serious levels and cause illness. Hands are a major source of infection spread. Although hand washing is effective in eliminating most microorganisms, there are circumstances in which this practice is not sufficient, and exposure justifies the use of an additional layer of protection. For these reasons, medical gloves are mandatory when performing invasive procedures or coming into contact with sterile sites [3]. 

According to World Health Organization (WHO) recommendations, protective gloves should always be used in cases of contact with blood, mucous membranes, injured skin, or other potentially infectious material, as well as hazardous chemicals and drugs [1]. The aim of this work is to review the materials used in medical gloves due to their importance as an element of personal protection. The purpose is to compare the natural and synthetic rubbers used in their manufacture as well as identify performance-enhancing materials that can be added to medical glove formulations to improve their properties. These materials include biopolymers, eco-friendly additives, bio-based fillers, and antimicrobial agents [4]. Similarly, we intend to address several prototypes of medical gloves, blends, composites, and coatings made from these new materials. 

### 1.1. History of Medical Gloves

Many healthcare workers were aware that accidental open lesions experienced while performing their duties could result in an infected wound, illness, and even death before the microbial nature of infection was established in the middle of the 19th century [5]. The exact time when protective gloves were first employed in the healthcare business is unknown. There are suggestions that an obstetrician called Walbaum covered his hands with sheep intestine as early as 1758 [6]. Other physicians used to cover their hands with cotton, silk, or leather gloves [5]. 

An important milestone in this field was the discovery of vulcanization by Charles Goodyear in 1839, when he was working at a rubber factory in Massachusetts and mixed a piece of rubber with sulfur on a hot stove [7,8]. He had discovered the vulcanization process, which turned natural rubber (NR) from a thermoplastic that could be softened by heat into a harder, more stable, and more durable product. Vulcanization consists of the development of a crosslinked rubber that is the product of the creation of bonds at several points of the individual NR chainlike molecules using sulfur as the crosslinking agent [9,10]. 

Vulcanized rubber quickly became the choice for coarse protective medical gloves. William Halsted of Johns Hopkins Hospital in Baltimore was likely one of the early promoters of sterile NR gloves in the operating room, but it is uncertain who initially encouraged their use. Halsted asked the Goodrich Rubber Company to make finer and less rudimentary NR gloves, although they were still quite stiff and difficult to handle. Over time, the NR gloves became even thinner and shorter. In 1897, the first article about sterile NR gloves in medical settings was published. This paper, entitled “Rubber gloves in the practice of surgery”, was written by Werner von Manteuffel and appeared in a German surgical journal [11]. By the beginning of the 20th century, the use of sterile NR gloves had become widespread in surgical practice [5].

### 1.2. Market of Medical Gloves

The rising incidence of epidemic diseases such as swine flu (H1N1) and the more recent and widespread COVID-19 (SARS-CoV-2) has driven the growth of the global medical glove market. As reported by the Financial Times, during the latter pandemic, glove industry sales and profits increased by over 100% [12,13]. According to data provided by Global Market Insights, the worldwide market of medical gloves grew dramatically as a result of the first phase of the COVID-19 pandemic expansion, reaching over USD 4 billion in 2020 [14]. In 2021, when the infection was best understood and the supply of these products increased in line with demand, this market experienced a slight decline in profits and reached USD 12.31 billion in value. Nevertheless, it is expected to increase at a compound annual growth rate (CAGR) of 5.8% from 2022 to 2030 [15]. 

**Figure 1:**
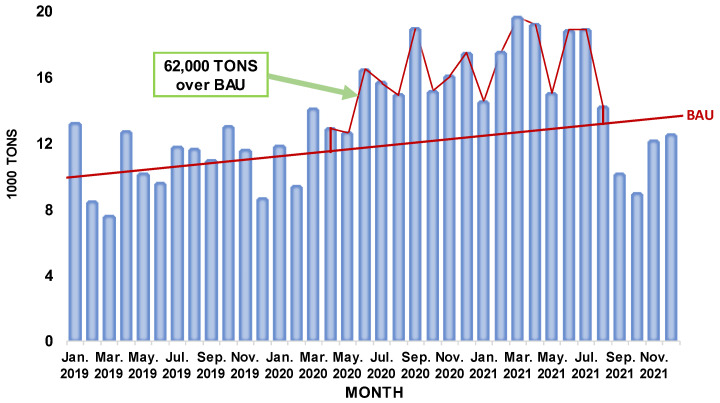
Imports of surgical gloves in the EU-27 from January 2019 to December 2021. Chart prepared by the authors based on Eurostat data [16].

Figure 1 shows EU-27 imports of surgical gloves between January 2019 and December 2021. The graph was compiled from the Eurostat dataset “DS-1180622” for product code: “B3-40151100 Surgical gloves, of vulcanized rubber other than hard rubber (excluding fingerstalls)”. In the graph, the business as usual (BAU) trend line was plotted using import data from January 2019 to March 2020, when the WHO proclaimed the global pandemic of COVID-19. To estimate the rise in medical glove imports during the COVID-19 pandemic, the over-BAU value was estimated using data from April 2020 to August 2021. The value of net imports in excess of BAU was approximately 62,000 Tons [16].

**Figure 2:**
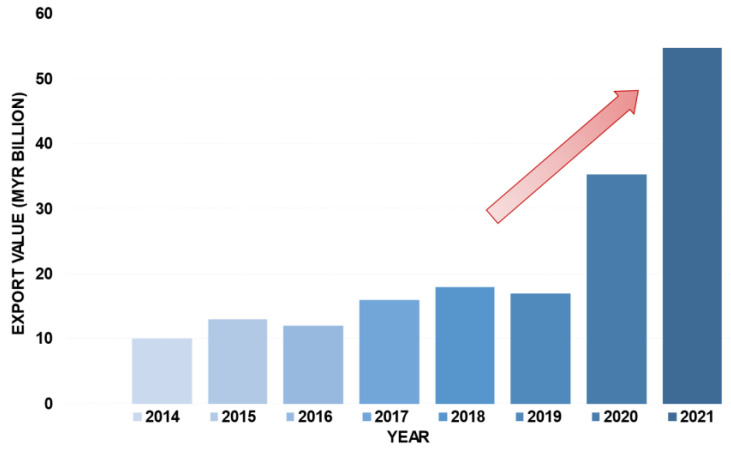
Exports of rubber gloves from Malaysia. The arrow indicates the sharp rise. Chart prepared by the authors based on MARGMA data shown in reference [18].

MARGMA (Malaysian Rubber Glove Manufacturers Association) estimates that the global demand for gloves grew by almost 200 billion units in the first months of 2020 due to the COVID-19 pandemic [17]. In 2021, at the peak of this pandemic, the global demand for rubber gloves reached 492 billion units. The exports of rubber gloves from Malaysia in monetary value terms from 2014 to 2021 are illustrated in Figure 2. This graph clearly reflects the significant growth that has occurred. Prior to the pandemic, the value of exports in Malaysian ringgit (MYR) did not exceed MYR 20 billion; however, by 2020, exports had reached MYR 35.26 billion, and in 2021, they peaked at around MYR 54.81 billion [18]. 

**Figure 3:**
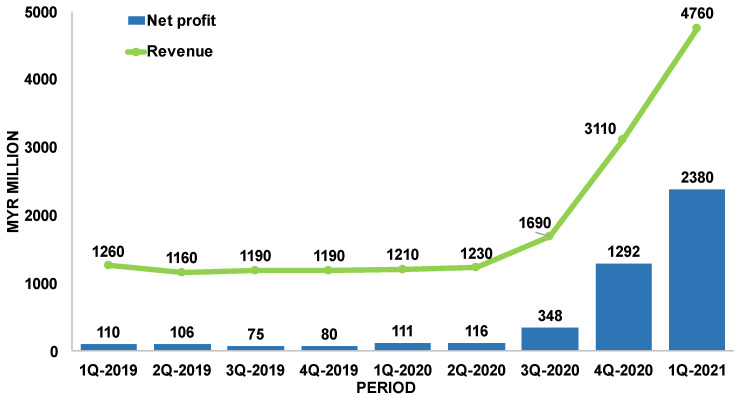
Quarterly financial report of Top Glove Corporation Berhad. Chart prepared by the authors based on Bursa Malaysia data shown in reference [21].

Major players in the glove market include Top Glove and Comfort Gloves [19]. Figure 3 shows the quarterly financial report of Top Glove Corporation Berhad with its earnings during the past pandemic period. In first quarter of 2021 (1Q-2021), this company achieved its maximum quarterly net profit of MYR 2.38 billion, and a high revenue of MYR 4.76 billion. The group’s quarterly net profit, compared to the previous quarter (4Q-2020), increased 84% from MYR 1.292 billion, while revenue increased 53% from MYR 3.11 billion [20,21]. 

**Figure 4:**
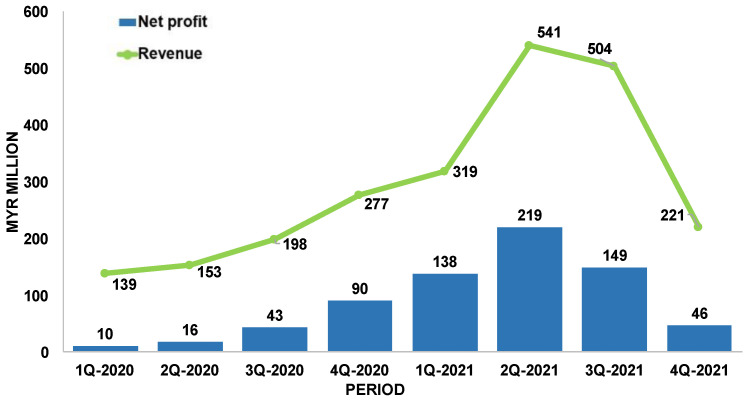
Quarterly financial report of Comfort Gloves Berhad. Chart prepared by the authors based on Bursa Malaysia data shown in reference [22].

The quarterly financial report of Comfort Gloves Berhad is presented in Figure 4. This chart shows that the revenue increased from MYR 138.65 million in 1Q-2020 (before COVID-19) to MYR 541.24 million in 2Q-2021, which represents a rise of 290%. In the same quarters, the group net profit amounts were MYR 10.24 million and MYR 219.13 million, respectively, which means an increase of 2040% [17,22,23]. 

In terms of the medical glove material market, natural rubber (NR) and acrylonitrile butadiene rubber (NBR) gloves are the most important sectors. NR gloves are the type that generates the highest revenues, due to their variety of applications in fields such as examinations and surgeries in the medical environment and as protection against chemicals and pathogens in the general industrial sector [24]. In the 2020 market share, the NR examination glove segment accounted for USD 5.1 billion, while the surgical glove segment reached USD 4 billion [14]. In 2021, the global NBR glove market was valued at USD 8.54 billion, and its size is expected to expand at a CAGR of 10.54% from 2022 to 2029. The NBR glove market attracted substantial new investments due to price incentives and increased demand resulting from the COVID-19 outbreak [25]. 

### 1.3. Production Process of Medical Gloves

**Figure 5:**
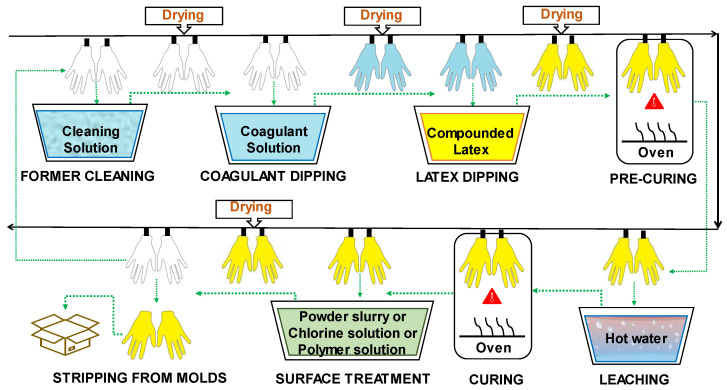
Production process of medical gloves by dipping.

The most common natural and synthetic rubber medical gloves are produced through the dipping process (Figure 5). Slowly, hand-shaped porcelain or metal molds are immersed in various tanks and subjected to different treatments. The main one is dipping in compounded latex, which consists of a mixture of natural or synthetic latex and compounding chemicals [26]. The compounding chemicals are the additives that must be included in medical glove formulations to achieve the required characteristics, such as mechanical strength, barrier integrity, color, aging protection, etc. [27]. These additives include vulcanizing agents, plasticizers, softeners, fillers, antioxidants, stabilizers, and different chemical compounds intended to improve processability [28,29].

The steps of the dipping process are briefly described below:

Former Cleaning: The procedure begins with washing and drying the hand-shaped molds. Alkaline solutions, acidic solutions, oxidizing agents, surfactants, and combinations of these can be employed as cleaning agents [28].

Coagulant Dipping: After cleaning the formers, they are coated with a coagulant, which is usually a polyvalent metal salt, an organic acid, or an organic acid salt [28]. The formers are dipped into the coagulant bath to promote adhesion and distribution of the compounded latex. The coagulant solution may also contain a separating agent, often calcium carbonate, which prevents the rubber from adhering to the molds. Subsequently, the molds are subjected to a drying process [26]. 

Latex Dipping: Next, the glove formers are dipped in a tank containing the compounded latex. The latter is a mixture of rubber suspension with several substances needed to form a glove, known as compounding chemicals. Formerly, the term “latex” referred to the white, milky sap gathered from the rubber tree; however, the terminology has also come to refer to dispersions of fine rubber particles in a liquid composed predominantly of water. Natural rubber (NR), polyisoprene rubber (IR), acrylonitrile butadiene rubber (NBR), and chloroprene rubber (CR) are mainly used in the dipping process [26]. 

Before adding other chemicals to commercial latexes, they must be stabilized to avoid alterations and variations in their ionic strength during the manufacturing process. The formulation ingredients must be integrated directly into an aqueous dispersion. For proper stabilization of the latex, the introduction of chemicals such as surfactants and rosin resins are required. Usually, two stabilization processes are needed; the provider performs the first stabilization step, but commercial latexes must be further stabilized before compounding chemicals are added. This second stabilization is mostly an electrostatic stabilization accomplished by altering the ionic strength of the latex. Table 1 presents some chemicals used for latex stabilization and their function [27].

Once the latex has been adequately stabilized, crosslinking agents are usually applied to bind the polymeric chains together and form a three-dimensional network that gives the material the desired flexibility and performance. The crosslinking process may involve the use of several crosslinking agents [27].

Vulcanization, in which crosslinking is carried out by means of sulfur bonds, is the most common technique [8]. Colloidal sulfur is often employed with NR, IR, and NBR latexes. Typically, 0.5 to 2.5 parts per hundred of rubber (phr) are used. Zinc oxide is utilized in the range of 4.0–5.0 phr for CR [26]. Carbamates in conjunction with thiazoles are ultra-fast accelerators for the crosslinking process. The latex mixture can alternatively be vulcanized by adding sulfur donors such as thiurams and thioureas as activators. Guanidines, or xanthates, also can be added [30].

Fillers, in particular calcium carbonate, are commonly used to reduce the cost of NR examination gloves [27]. The degree of reinforcement offered by a filler for a rubber glove depends on many factors. The most crucial aspect is to achieve a large filler–rubber interface, which only colloidal filler particles can offer. To avoid dispersibility and processability concerns, the particles must have a specific surface area between 6 and 400 m^2^/cm^3^ [31]. 

Medical gloves contain antioxidants that defend them against attack by oxygen while in storage. Surgical and examination gloves contain non-staining antioxidants such as phenolic antioxidants (styrenated and hindered phenols), which are sometimes combined with a secondary antioxidant [30].

Pigments and dyes are combined with gloves to achieve opacification and impart the desired hue to the product [27]. The use of pigments or UV absorbers can improve light fastness to prevent hardening of NR gloves when exposed to direct sunlight. Also, by adding so-called antiozonants, protection against ozone can be accomplished [30].

Pre-curing: After the latex dipping process, another drying phase takes place. In this stage, the curing process is partially carried out, which is called the pre-curing process. The compounded latex that has been deposited on the molds is allowed to acquire a certain wet gel strength before the leaching step [28].

Leaching: This stage is often referred to as “wet gel leaching.” Once the latex mixture has dried, residual chemicals and proteins on the gloves surface are removed through immersion in tanks of hot water. The tanks are refilled periodically with fresh hot water [28]. The water immersion period ranges from 1 to 10 min, depending on the film width. Washing NR latex film in a weak aqueous alkaline solution, such as aqueous ammonia or aqueous potassium hydroxide solution, facilitates protein removal [26].

Curing: This process, also simply referred to as vulcanization, often involves a hot-air circulation blower. The lowest vulcanization temperature varies depending on the compounded latex. Normal ranges for NR and IR are 90–100 °C, for NBR 120–140 °C, and for CR 120–130 °C [26]. The rubber reaches its final strength upon leaving the vulcanization oven [28]. 

Surface treatment: The purpose of the treatment of the inner surface of gloves is to prevent sticking together, to facilitate donning, to ensure a smooth fit, and to provide comfort during use. Traditionally, powder was employed for this purpose. However, powder was associated with increased risks of irritation or hypersensitivity for both users and patients, especially in NR gloves. NR latex proteins, which cause allergies, adhere to the powder, and spread rapidly in the environment, increasing the prevalence of allergies. As a result, the use of powder is increasingly restricted by regulation. In several countries, such as the United States, Germany, and the United Kingdom, powder is prohibited [27,32]. As an alternative, other treatments can be applied, such as chlorination and polymeric coatings [33].

Powdered gloves are formed by dipping them in a slurry. This substance is also known as wet powder, and contains talc, silica, or crosslinked starch. For the chlorination process, the gloves are dipped in a solution containing chlorine. The reaction with the chlorine forms a thin film of chlorinated rubber on the glove surface. The chlorine solution is produced by pumping chlorine gas into the water or by combining hydrochloric acid with sodium hypochlorite [26]. Probably the most widely used method for producing powder-free NR gloves is chlorination. The double bonds of the polymer chains present in NR are highly prone to the addition of chlorine, which has the effect of stiffening and detackifying the rubber surface of the glove [28]. 

Regarding polymer coating, it is common practice to dip gloves in hydrogel, an aqueous dispersion based on acrylic or polyurethane diluted to the required concentration, silicone polymer, or a polymer blend [26]. Coatings can be classified into two categories: hydrogels and non-hydrogels. Hydrogel coatings are composed of substances that absorb water several times their weight, swell, and become slick so that gloves can be easily donned. Non-hydrogels are water-repellent, and the coating’s topology matches the features of a powdered surface. Often, a dual strategy is employed: first, the donning side of the glove is coated, and then the grip side is chlorinated [28].

Stripping from molds: After surface treatment, the gloves undergo a drying process and are then demolded and packaged for sale [26].

### 1.4. Environmental Concerns Related to Medical Gloves

The global demand for rubber gloves keeps increasing despite the environmental problems related to their disposal [34]. Rubber gloves account for 24% of total medical solid waste [35]. Discarded NR gloves typically take at least two years to degrade in a natural environment. Many highly additivated and crosslinked commercial NR gloves require even longer to fully decompose in soil under ambient conditions [36].

The various stages of rubber glove production require multiple resources, including potable water, chemicals, energy, and electricity. Water is often used for the preparation of the compounded latex, as well as for cleaning, leaching, and cooling procedures. Heat is utilized in the drying and curing processes. Electricity is mainly used for lighting, pumping water, operating heavy machinery, and the treatment of liquid waste [37].

At each stage of the glove manufacturing process, there are material inflows and waste outflows. Contaminated rinse water flows can be said to occur throughout the washing and leaching stages. In operations involving heating or mechanical action, energy is consumed. Ovens fueled by liquefied petroleum gas (LPG) produce carbon dioxide emissions as well as energy losses. Gloves and packaging materials are also discarded downstream in the production process. This manufacturing technique has effects on the environment as well as human wellness [38].

It is important to note that sulfur is one of the most widely used crosslinking agents. The sulfur-based curing system (vulcanization) is harmful from the point of view of environmental and health problems. The emission of toxic sulfur-based gases can cause acid rain, which returns considerable quantities of sulfuric acid to the earth, destroying vegetation and degrading soil quality. In addition, gaseous sulfur compounds can induce irritation and inflammation of the respiratory system. Higher levels of sulfur dioxide can cause eye burns and be fatal to humans [39]. In addition, accelerators such as benzothiazoles, which are toxic to aquatic life, are used in the vulcanization process [40]. 

To counterbalance the disadvantages of the traditional sulfur process, alternative curing methods include metal ionic crosslinkers, organic peroxides, or physical methods such as UV and gamma rays. The basic mechanism underlying the functionality of the metal ion as a crosslinker is related to its charges. Sulfur forms covalent bonds between elastomer chains in vulcanization, and these sulfur bonds can be replaced by an ionic bond with a multivalent metal ion, resulting in a reduction in process time and energy consumption. The most common applications of metal ion crosslinking are NBR and CR gloves. As this method does not require initiators or crosslinking accelerators, the cost of materials is reduced [39]. 

In ultraviolet (UV) crosslinking, covalent bonds are generated via the UV-assisted thiol–ene reaction, which represents an unconventional method for the crosslinking of NR. It can be carried out at room temperature with short process times and without the use of hazardous chemicals. UV-crosslinked NR articles exhibit good skin compatibility and high tensile strength. Both the lattice density and Young’s modulus have been found to increase with radiation intensity [41]. 

With respect to gamma ray crosslinking, research has shown that carboxylated NBR can be crosslinked (forming covalent bonds) through high-energy radiation, such as gamma rays or electron beams [41]. The advantages of this procedure include the absence of hazardous chemical residues, full control of the crosslinking density, and improved mechanical properties of the crosslinked material. Disadvantages include the large amount of energy required for the process, the fact that direct exposure of humans could cause cancer, and the lack of available technical data [42]. 

In recent years, the widespread usage of rubber and the resulting large amount of waste of this material has increased interest in this field, with the objective of applying bioremediation. NR can be degraded by bacteria and fungi, but the process is slow and even slower in gloves with higher crosslinking densities [35,43]. Linos et al. (2000) found that *Pseudomonas aeruginosa* AL98, a type of Gram-negative bacterium, was capable of disintegrating NR, in its natural form as NR latex concentrates or in its crosslinked forms as NR or IR gloves [44]. 

Although the biodegradation of NR has been widely investigated, progress in this field of study has been hampered by the difficult isolation of appropriate bacteria, extended cultivation periods, and the scarcity of genetic tools [45]. *Actinomycetes* have dominated the literature about the rupture of *cis*-1,4-polyisoprene among NR-degrading bacteria. The most prominent genera are *Streptomyces*, *Mycobacterium*, *Nocardia*, and *Gordonia* [46]. The three latter species directly attack the NR substrate, producing a biofilm and fusing with the polymer to induce cell surface degradation. The adherent group of bacteria has been implicated as much more efficient degraders of this substance than enzyme-secreting strains [47]. 

There is evidence that some NR glove additives limit microbial descomposing action. It has been demonstrated that the extraction of these inhibitory substances (antioxidants) using organic solvents promotes the proliferation of *Gordonia* and *Micromonospora* species. However, using chemical solvents to remove rubber inhibitors is not environmentally friendly, so an alternative via microbial action was studied. Due to the similarities between rubber additives and fungal degradable chemicals, the successful cleavage of antioxidants by white rot fungus has been reported [46].

**Figure 6:**
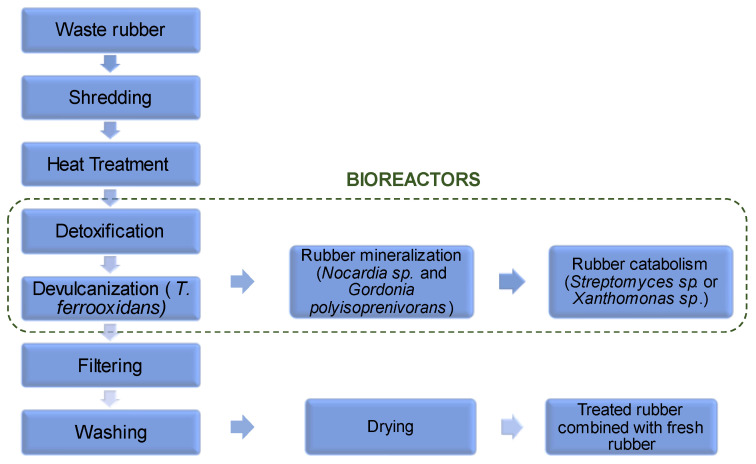
Recycling and remediation of NR through microbial action. Reprinted and adapted with permission from reference [46]. Copyright © 2013 Springer Nature.

An example of a plant for the recycling and remediation of NR by microbial action is shown in Figure 6. The waste NR is ground to promote further microbial attack. The ground rubber is then heated to denature the unstable compounds, while sterilizing the rubber to ensure the absence of pathogenic species that could inactivate or compete with the microorganisms used in the bioreactors [46]. 

After heating, a detoxification process is performed in which white rot fungi can be used to degrade the NR additives. Once the additives have been removed, a devulcanization process is performed with *Thiobacillus ferrooxidans* to break the sulfur bonds of the NR. The decomposition can be completed with potent degrader agents such as *Nocardia* sp. and *Gordonia polyisoprenivorans*. Then, the lower-molecular weight molecules can be catabolized by *Streptomyces* sp. or *Xanthomonas* sp. Alternatively, the devulcanized NR can be filtered, cleaned, dried, and blended with fresh NR for reprocessing [46].

## 2. Types of Medical Gloves

There is a variety of medical gloves based on the specific requirements of each application. Essentially, the two main types of medical gloves are examination gloves, used for normal medical check-ups and minor operations, and surgical gloves, used for operations [48]. Examination gloves are thin (50–150 µm) and ambidextrous. As they are usually for short-term use, they can be sterile or non-sterile depending on the risk to be handled. On the other hand, surgical gloves are always packed in a sterile bag in pairs, distinguishing the right hand from the left. These gloves are thicker than examination gloves (180–250 µm) as they are worn longer; it is advisable to change them every 90 min, or less if a perforation is detected [27]. 

**Figure 7:**
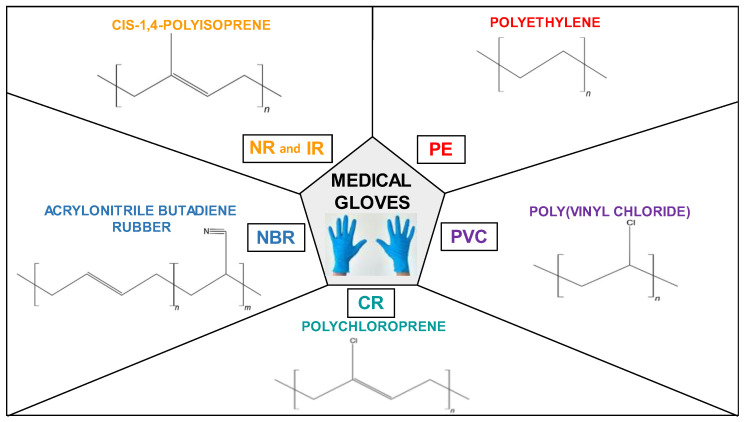
Chemical structure of common types of medical gloves.

The most common types of medical gloves (Figure 7) include those made of the following materials: natural rubber (NR), polyisoprene (IR), acrylonitrile butadiene rubber (NBR), chloroprene (CR), polyethylene (PE), and poly(vinyl chloride) (PVC) [32].

### 2.1. Natural Rubber (NR)

Natural rubber (NR) is a key raw material that has modernized the world due to its wide functionality and excellent elastic properties. NR is present in the latex of more than 2000 plant species, including *Hevea* sp., *Castilla* sp., *Manihot* sp., *Guayule* sp., and *Taraxacum kok-saghyz* sp. [27,49]. Surprising examples, such as dandelions, are included. However, only one tree source, *Hevea brasiliensis*, is commercially significant [31]. 

*Hevea brasiliensis* NR latex is a colloidal system of *cis*-1,4-polyisoprene particles dispersed in an aqueous serum. The milky white sap consists of approximately 34% *cis*-1,4-polyisoprene, 2–3% protein, 0.1–0.5% sterol glycosides, 1.5–3.5% resins, 0.5–1.0% ash, 1.0–2.0% sugars, and 55–65% water [31,50]. The production of milky latex generated by the *Hevea brasiliensis* tree fluctuates between 19.8 g and 90.5 g per tree and per tap, using a half-spiral cut extraction method on the bark of the tree with alternating daily harvesting [42].

NR gloves, also known as latex gloves, are made of 90% to 95% NR and 5% to 10% compounding additives [30]. Thus, NR gloves are waterproof, and they exhibit excellent mechanical properties, such as high elasticity, tactility, and tension retention [39]. These gloves are excellent for delicate applications due to their extreme comfort and sensitivity [51]. The minimum and maximum operating temperatures are −51 °C and 104 °C, respectively. Most medical examination and surgical gloves are made of this material, which provides excellent barrier protection against microorganisms and infectious fluids [42]. A negative aspect of NR is the presence of impurities such as proteins, which have antimicrobial properties and play an important role in plant defense responses, but whose remaining presence in NR gloves causes allergies to a certain part of the exposed population [52,53]. Sensitization may occur with repeated exposure [54]. NR gloves typically have extractable protein (EP) levels ranging from 20 to 1000 μg/g. Despite this, EP can be removed through various leaching processes [1]. Once NR gloves were identified as a source of allergen exposure, awareness was raised, and risk reduction measures were implemented. The transition to powder-free, low-protein NR gloves and synthetic gloves corresponded with a decrease in the incidence of allergies [55]. 

### 2.2. Polyisoprene (IR)

Polyisoprene rubber (IR) is a synthetic rubber with the same chemical composition as NR and therefore shares similar properties. Shell Company was the first to commercialize IR in 1960 [56]. IR has a more uniform and lighter color than NR. IR also has a higher tensile and tear strength due to a narrower molecular weight dispersion. This material behaves like NR during processing and can be crosslinked using the same techniques [31]. Most synthetic surgical gloves are made of IR and are characterized by their high dexterity, sensitivity, absence of protein, and high level of wearer comfort [57]. IR contains 90–92% *cis*-1,4-polyisoprene, while NR contains approximately 99% of this configuration [58].

### 2.3. Acrylonitrile Butadiene Rubber (NBR) 

Acrylonitrile butadiene rubber (NBR), also known as nitrile rubber, was patented in 1934 by the chemists Erich Konrad and Eduard Tschunkur of IG Farabenindustrie [59]. The acrylonitrile content (18% to 50%) in this material gives it higher hardness, higher resistance to oil and non-polar solvents, and better puncture and abrasion resistance compared to NR [31]. This material is used in various surgical and examination gloves. They are usually blue, purple, or black, and any needle puncture is evident [32]. NBR gloves have a longer shelf life than NR gloves [24]. NBR gloves are flexible, soft, and comfortable. However, they have drawbacks such as lower sensitivity and rougher texture than NR gloves [51]. Their pathogen protection and temperature tolerance are moderate, with lowest and maximum working temperatures of −34 °C and 121 °C, respectively [42]. NBR is one of the most widely used synthetic rubbers because of its lower cost compared to other synthetic rubbers [39,51].

### 2.4. Polychloroprene (CR)

Polychloroprene (CR) is a DuPont patented and registered product known as Neoprene^®^ [42]. It is produced through emulsion polymerization of chloroprene [31]. This is one of the most frequently used synthetic rubbers for making gloves that are resistant to both temperature and aggressive chemicals. Its environmental resistance, thermal stability, and good oil resistance make it a standout in the glove sector [39].

CR gloves fit and feel like NR gloves. They are very comfortable and suitable for people sensitive to NR. These gloves are extremely durable and can stretch quickly while maintaining their original shape due to their high elasticity [60]. Their mechanical and flammability resistance are also superior to those of NBR gloves [42]. The minimum and maximum operating temperatures are −25 °C and 93 °C, respectively [61].

### 2.5. Polyethylene (PE)

Polyethylene (PE) is a polymer synthesized through polycondensation of ethylene. PE is malleable, flexible, and resistant to heat, electrical current, chemicals, and degradation [62]. Thin PE foils are welded together to create PE gloves available in various thicknesses and with textured surfaces. They have a wide range of applications, including non-sterile medical work, food handling, painting, and handling of electronic components. The protective effect depends more on the strength of the welded seams than on the inherent chemical resistance of the material [30]. 

### 2.6. Poly(vinyl Chloride) (PVC)

Polyvinyl chloride (PVC) is a synthetic rigid polymer that was converted into a flexible material by Waldo Semon at BFGoodrich in the 1920s. Flexible PVC is vinyl compounded with a plasticizer, which defines the properties of the final product [63]. Traditionally, phthalates have been added to PVC as plasticizers. These substances have been gradually replaced with less harmful substitutes such as adipates and vegetable oils [64]. PVC gloves, also known as vinyl gloves, are stiffer than NR gloves and have comparatively lower elastic modulus, tear strength, tensile strength, feel, and comfort, but on the plus side, they have no residual protein and are less expensive [27,65]. PVC gloves are usually transparent and fit loosely; they can be used in non-sterile environments and for handling non-hazardous materials and drugs [32]. PVC gloves are permeable; investigations into the permeability of gloves exposed to 13 chemotherapeutic drugs indicated that even after short-term applications, transfer to the wearer’s skin occurs [51,66]. These gloves are easily worn out by use [67].

Table 2 summarizes the main advantages and disadvantages of the different types of medical gloves.

## 3. Mechanical Properties of Medical Gloves

There are international requirements that must be followed for medical gloves to be suitable for their intended purpose. As an example, the ASTM standards for NR, NBR and CR rubber examination gloves are presented in Table 3. 

The ASTM D3578 – 19 specification dictates the mechanical property values that NR examination gloves must reach. The appendix of the standard provides physical criteria for Type I and Type II gloves. This classification has been extended to provide customers with a greater selection of fit, feel, and comfort [68]. For NBR and CR examination gloves, the mechanical property values are dictated by ASTM D6319 – 19 [69] and ASTM D6977-19 [70], respectively. 

For NR, NBR, and CR examination gloves, one of the following accelerated aging tests must be performed: (a) being exposed to 70 ± 2 °C for 166 ± 2 h or (b) 100 ± 2 °C for 22 ± 0.3 h. Aging tests are designed to demonstrate that the performance of the gloves will not deteriorate before the date of expiry. Accelerated aging testing is required since it is impracticable to conduct real-time aging tests prior to releasing these products onto the market. Under the standards’ test conditions, gloves must be able to resist the deterioration caused by oxidative and thermal aging. Mechanical properties are expected to be altered over the lifespan of the product, so they are measured before and after the aging test to verify that gloves keep their physical integrity and protective capability [68,69,70]. 

The specifications for the mechanical properties of medical gloves according to European standards are addressed in EN 455-2:2015 (Medical gloves for single use. Part 2: Requirements and testing for physical properties). For accelerated aging, the gloves are heated in an oven at 70 ± 2 °C. The minimum force at break (before and after aging) for surgical gloves must be 9.0 N, for examination gloves except for thermoplastic materials 6.0 N, and for examination gloves made of thermoplastic materials (e.g., PVC, PE) 3.6 N [71].

Table 4 shows the mechanical properties of examples of gloves made of NR and NBR of KOSSAN Rubber Industries gloves published on the company website. It can be seen that the properties of the products meet the normative requirements [72]. 

## 4. Prototypes of Medical Gloves with Performance-Enhancing Materials

The long-term viability of medical glove manufacturing processes is crucial from both a financial and environmental protection point of view. The use of performance-enhancing materials such as biomaterials, bio-fillers, biodegradable polymers, antimicrobial agents, etc. in conjunction with natural and synthetic rubbers could help to support the three pillars of sustainability in the environmental, social, and financial sectors. 

Biomaterials such as bio-fillers help accelerate gloves’ degradation after disposal. Thus, the extraction of bio-based chemicals and their incorporation into the polymeric matrix could lead the way in a new era in disposable glove manufacturing [73]. Food waste, terrestrial vegetation, and aquatic plants such as micro and macro algae could all be sources for these bio-based compounds [39]. Since the green market is growing dramatically each year, the introduction of biodegradable rubber gloves onto the market within the green technology sector would present an opportunity for manufacturing companies [36].

Antibacterial components have become prevalent in daily life, and the antibacterial properties of nanoparticles are rapidly being investigated and commercialized [39]. Despite being sterilized and separately packed, surgical gloves are exposed to germs when the package is opened [74].

The increasing number of antibiotic-resistant microorganisms has led to the search for new agents that can prevent the spread of pathogenic microorganisms. Antibacterial agents with the potential to be incorporated into natural or synthetic rubber gloves include biguanides such as chlorhexidine salts and poly(hexamethyl biguanide) (PHMB), quaternary ammonium salts such as benzalkonium chloride and benzethonium chloride, chlorinated phenols such as triclosan, essential oils such as farnesol, phenoxyethanol, octoxyglycerin, antifungal agents, iodine compounds, silver salts, some vegetable oil extracts, such as gentian violet, brilliant green, chitosan-based compounds, turmeric, and similar substances [4]. By covalently bonding the antibacterial agent to polymer surfaces, it is feasible to achieve an enduring effect, which leads to self-sterilized materials that may protect themselves from pathogens and contribute to the eradication of harmful microbes [75].

### 4.1. Biodegradable Green Gloves Containing Ascorbic Acid from Maleate Epoxidized Natural Rubber/Poly(vinyl Alcohol) Blend

Poly(vinyl alcohol) (PVA) is a biodegradable polymer that has been used as precursor material for the production of decomposable gloves, as it is non-toxic, physically and chemically resistant, and economically viable. Previous research has reported the improvement of biodegradability when PVA is combined with NR [76]. Ascorbic acid (L-ascorbic acid), also identified as vitamin C, has been shown in numerous studies to have antibacterial properties. It has been demonstrated that it inhibits the growth of *Helicobacter pylori*, *Campylobacter jejuni* [77], *Staphylococcus aureus*, *Enterococcus faecalis* [78], and *Mycobacterium tuberculosis* [79]. In vitro studies have demonstrated that L-ascorbic acid can improve the action of antibiotics like azithromycin [80] and levofloxacin [81,82]. 

**Figure 8:**
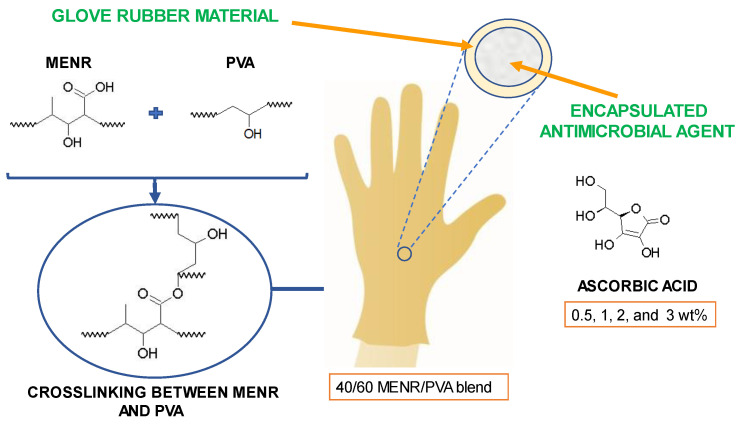
MENR/PVA blend glove with encapsulated AA. Graphic prepared by the authors based on reference information [83].

Riyajan et al. studied maleate epoxidized natural rubber (MENR) and PVA (MENR/PVA) blends for producing a biodegradable glove with ascorbic acid (AA) encapsulated, represented in Figure 8. To produce MENR, under a nitrogen atmosphere and intensive stirring, 20 wt% NR latex was combined with 10% non-ionic surfactant. Then, formic acid and water were added to the previous mixture, which was held at 30 °C for 15 min. The temperature was increased to 70 °C and the reaction was completed after 5 h of stirring. Maleic anhydride (MA) in the presence of 10% Triton X-100 was then added to the resultant epoxidized natural rubber (ENR) latex at 80 °C and agitated for 3 h. The mixture was agitated for 15 min at 70 °C, after the addition of the free radical initiator potassium persulfate [83]. 

To prepare the MENR/PVA blends for the gloves, a 10 wt% PVA aqueous solution was combined, at 78 °C, with various MENR concentrations of 10, 20, 30, and 40% using magnetic agitation. Then, on glass plates, 80 g of the MENR/PVA blends was dehydrated at 30 °C for 3 days. The biodegradation of this material was examined by monitoring the weight loss of samples with different proportions of PVA and MENR in the blend. Samples of PVA alone and with MENR contents of 10, 20, 30, and 40% blended with the PVA were evaluated. The samples were weighed and then buried in soil, irrigated daily with water to maintain its moisture content, at ambient temperature. PVA alone exhibited the greatest biodegradation, due to the existence of hydroxyl groups in this compound. After 10 days buried in the soil, 50% of the PVA’s weight had been lost, and after 40 days, it had decomposed completely. The biodegradation rate of the samples is reduced as the MENR proportion in the sample is increased, because crosslinking takes place. Nevertheless, all the blends decomposed properly in the natural environment through water-induced hydrolysis and enzymatic breakdown. After 90 days, at the end of the experiment, the samples containing 10 and 20% of MENR almost had a weight loss of 100%, and the samples with 30 and 40% of MENR lost around 75 and 60% of their weight, respectively [83].

The encapsulation of AA in a 40/60 MENR/PVA blend was explored in order to impart antibacterial activity to gloves. The encapsulation efficiency (EE) was 100, 99, 98.5, and 96%, respectively, for 0.5, 1, 2, and 3 wt% AA. The cumulative in vitro release of AA from the MENR/PVA blend films can be described as two distinct stages based on these data. The first 12 h are characterized by a burst release phase in which about 25, 33, 38, and 43% of the total AA was released from the MENR/PVA blends with 0.5, 1, 2, and 3 wt% AA, respectively. During this phase, AA was released via diffusion through the walls of the MENR/PVA blend. Up to 70 days, the release process is characterized by a more progressive release, accounting for approximately 100, 90, 75, and 64% of the total for 3, 2, 1, and 0.5 wt% AA, respectively. The initial burst release is caused by the leaching of AA near the capsule walls. As there is no polymer coating, the rate of matrix dissolution is quite rapid, and AA adjacent to the wall could promptly diffuse away [83]. 

It has been established that gloves manufactured with an MENR/PVA blend containing antimicrobial agent effectively prevent microbial transmission. Controlled and optimized release of AA from the MENR/PVA blend could play a significant role in the development of a medical glove [83].

### 4.2. NR Films/Gloves and Carboxylated-NBR (XNBR) Films Containing Sago Starch as Bio-Filler

The creation of effective bio-based products would aid in the prevention of environmental degradation. NR can be utilized as a matrix material in composite applications, where it is supplemented with bio-fillers to improve thermo-mechanical and barrier properties. In NR gloves, efforts to substitute ordinary calcium carbonate with bio-fillers such as polysaccharides, eggshell, and chitosan are frequently considered [36]. The advantages of employing bio-fillers over synthetic fillers are their renewability, abundance, and low cost; the negatives are comparatively weaker mechanical qualities. Because cellulose, chitin, and starch are hydrophilic, they are less compatible with the NR matrix. Achieving a homogenous filler–NR matrix mixing is difficult due to the different structural features of the components. Fillers with small particle size enhance the physical interaction with the matrix. Hence, the mechanical resistance, thermal stability, sorption, crystallinity, and biodegradability of the bio-fillers can be improved as result of their smaller size. On the other hand, the presence of hydroxyl groups in bio-fillers may result in low compatibility with NR [84]. 

A chemical treatment of the bio-filler can reduce the hydroxyl group content to improve compatibility, resulting in composites with higher strength and crystallinity. Further research is required to investigate the primary obstacles: inadequate hardness, moisture absorption, and suitability for outdoor and heavy-duty uses [85]. It is known that some bacteria and fungi are capable of degrading NR, despite the lengthy nature of the process [86]. The addition of polysaccharides to the NR system serves to enhance the action of microorganisms, facilitating degradation via enzymatic polysaccharide rupture and oxidation of the rubber backbone chain [87]. The polysaccharides are particularly favorable for the biodegradation process since they can be used as sustenance for microorganisms, hence promoting their proliferation and degradative action [36]. Starch is a typical polysaccharide used in biodegradable rubber films. It is made up of 70–80% amylopectin and 20–30% amylose [88].

Amylose content is a key criterion for its usage as a biodegradable material since it may provide nutrients to microorganisms, allowing them to begin the biodegradation activity [34]. When compared to other forms of starches, sago palm (*Metroxylon sagu*) starch has a greater amylose concentration (27%). To reach the required qualities of rubber films, starch must undergo a physical or chemical transformation. Acid hydrolysis may be used to chemically modify native sago starch (NSS) by inducing the creation of sulphate ester groups on the starch surface, which increases the interaction between the rubber matrix and the starch [89].

**Figure 9:**
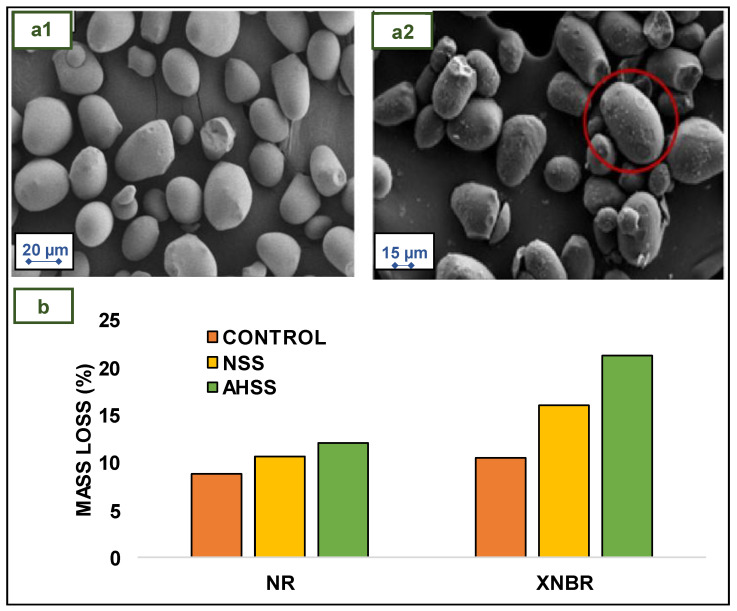
SEM images of (**a1**) NSS and (**a2**) AHSS. The red circle shows the porous surface of the starch particle after acid hydrolysis (**b**) Mass loss of NR and XNBR films (control, NSS-filled, and AHSS-filled) after 3 weeks. Reprinted and adapted with permission from reference [34]. Copyright © 2019 Elsevier.

Daud et al. designed an experiment with sago starch to improve the biodegradability of NR and XNBR films. Sago starch with sulphate ester groups (AHSS) was obtained by treating NSS with aqueous sulfuric acid solution for 7 days at room temperature. The particle size of NSS was initially 1.233 µm, and it was lowered to 0.313 µm after the acid hydrolysis process. SEM micrographs of the NSS and AHSS are shown in Figure 9(a1) and Figure 9(a2), respectively. The surface of the AHSS particles is more porous, more rugged, and largely eroded than that of the NSS particles. In order to make an adequate comparison, unfilled NR, NSS-filled NR, AHSS-filled NR, unfilled XNBR, NSS-filled XNBR, and AHSS-filled XNBR films were prepared. To prepare the films, NR latex was mixed with compounding ingredients (with or without filler, depending on the case) and mechanically stirred for 1 h to obtain the NR compounded latex, which was then matured for 24 h at room temperature prior to the dipping process. For the prevulcanization procedure, the NR compounded latex was then heated to 80 °C and continuously stirred. XNBR latex was compounded similarly to NR latex, with the difference that the maturation period was 48 h. Prior to the dipping procedure, the prevulcanized compounded latexes were stirred for 15 min. Clean aluminum plates were dipped for 10 s in a coagulant bath, dried for 5 min, and left to cool at room temperature for 5 min before being dipped for 10 s in a latex dipping tank and cured at 100 °C. NR and XNBR were cured for 10 and 90 min, respectively [34]. 

Regarding mechanical behavior, in both cases, NR and XNBR unfilled films have the best properties. The poor interfacial bond between the hydrophilic sago starch and the hydrophobic rubbers resulted in a decrease in the tensile properties of films when NSS was added. Incorporating AHSS into the films improved the mechanical properties and swelling resistance of NR and XNBR compared to NSS. This distinction may be attributed to the superior compatibility of AHSS in NR and XNBR films compared to NSS in these same films. The low amorphous content, reduced particle size, and existence of sulphate ester groups contribute to the increased rubber–filler interaction between AHSS and the rubber matrix [34]. 

Figure 9b shows the mass loss of unfilled, NSS-filled, and AHSS-filled NR and XNBR films after 3 weeks of soil burial. The percentage of mass loss was highest for AHSS-filled NR films, followed by NSS and unfilled NR films [34]. 

The mass loss tendency of NR films is comparable to that of XNBR films. Both the unfilled NR and XNBR films experienced a lower mass loss. Compound additives, such as sulfur, are reported to inhibit the rate of biodegradation of rubber films. Incorporating sago starch, however, would encourage soil microorganisms to consume this bio-filler and secrete enzymes that can degrade rubber molecular chains [36]. The AHSS-filled NR and XNBR films showed significant mass loss. This could be accredited to the decrease in the amorphous section after acid hydrolysis of sago starch, which makes rubber and glycosidic chains more susceptible to attack by microorganisms [34].

Rahman et al. studied the degradation of gloves made from NR with sago starch as bio-filler from buried soil samples by a mixed culture containing starch-degrading bacteria as well as NR-degrading bacteria. The aim of the starch hydrolysis test was to confirm the presence of starch-degrading bacteria in the mixed culture. In this test, the evaluated bacteria were grown on agar plates containing starch. After incubation, an iodine indicator was added to the plates. Hence, when a few drops of potassium iodide solution were applied to the sample, the surface of the plate became blue-black because the reaction between starch and iodine produces polyiodide chains. The amylose in starch forms helices around which the iodine molecules are clustered. This blue-black color does not occur when starch is broken down or hydrolyzed into smaller carbohydrate units. Therefore, transparent, clear zones were formed next to the colonies that hydrolyze starch, while the other parts of the plate remained colored [90]. 

**Figure 10:**
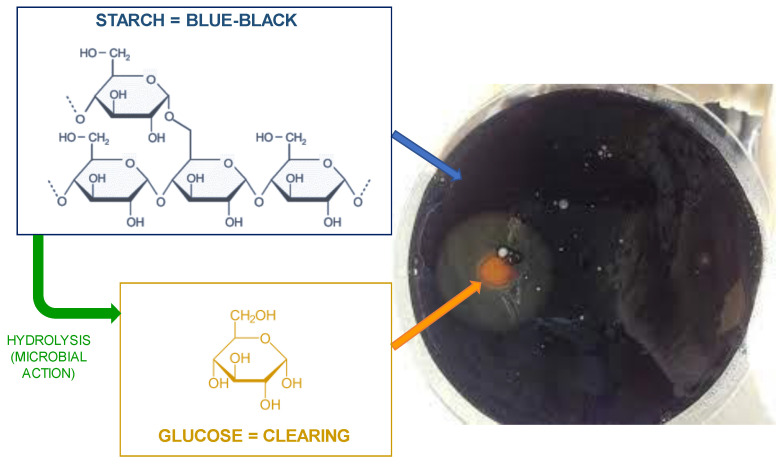
Starch hydrolysis test of the mixture culture. The blue and the orange arrows show the areas where the starch remains unchanged and where it has been hydrolyzed to glucose by microbial action, respectively. Reprinted and adapted with permission under a Creative Commons license (CC BY 3.0) from reference [35].

Figure 10 shows a clear zone in the iodine test on an agar plate that proved the starch hydrolyzation. Based on the biodegradation rate data, the presence of starch-degrading microorganisms as well as rubber-degrading bacteria was detected, which accelerated the biodegradation of sago-filled NR gloves by 53.68%, while the biodegradation rate for NR gloves (without filler) was lower, at around 50.31% [35]. 

### 4.3. Mangosteen Peel as Antimicrobial Agent in NR Gloves

Xanthones are secondary metabolites found in plants, fungi, and lichens. They have been isolated in the pericarp area of the mangosteen, a typical fruit of the tropics. Xanthones have potent antioxidant, anticancer, anti-inflammatory, anti-allergic, antibacterial, antifungal, and antiviral properties [91]. In fact, the peel of mangosteen is a kind of hydrophobic biomaterial that can be used in medical care, cleaning products, skin care, and cosmetics. It can inhibit exposed cells such as *S. aureus*, *S. albus*, and *M. luteus*, as well as plant pathogenic fungus like *F. oxysporum f.* sp. *vasinfectum*, *A. tenuis*, and *D. oryzae*. It is also effective against *P. acnes* and *S. epidermidis* and can be used as an alternate therapy against acne. Furthermore, due to proven good properties, it can suppress cancer cells and has potential for both preventative and therapeutic purposes [92]. 

Moopayak and Tangboriboon used mangosteen peel as a bio-filler to produce NR medical gloves. The addition of mangosteen peel powder to NR formulation as a bio-filler can improve the antimicrobial properties of the gloves without sacrificing the softness, film thickness, and mechanical characteristics [93].

**Figure 11:**
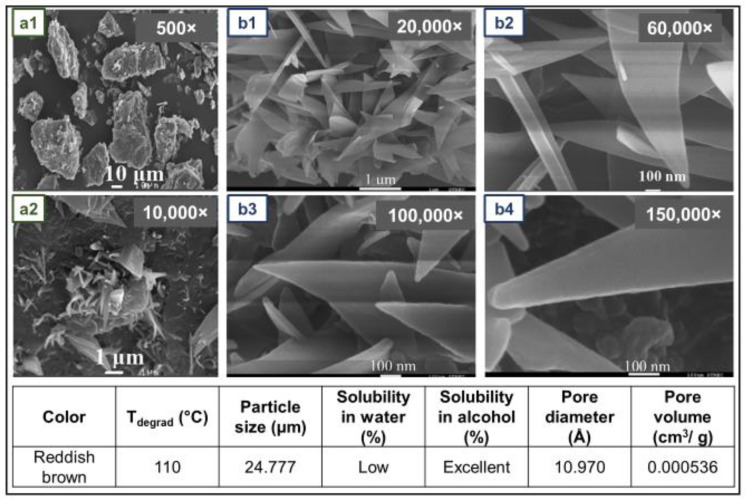
SEM (**a1**,**a2**) and FESEM (**b1**–**b4**) micrographs of mangosteen peel. The main physical properties of mangosteen peel powder are summarized below. Reprinted and adapted with permission from reference [93]. Copyright © 2020 John Wiley and Sons.

NR gloves with mangosteen peel powder have been obtained with good appearance, smooth, transparent, and thin, with good elongation, good tensile strength, no water leakage, and no skin toxicity. Comparing NR gloves with and without mangosteen peel, it was detected that the mechanical properties with the addition of the bio-filler were not only preserved, but slightly improved. The microstructure of the mangosteen peel used is presented Figure 11 [93].

To prepare the NR gloves, a porcelain hand mold, concentrated NR latex, and compounding chemicals were used. The mold was washed, dried, and dipped for 3 s in coagulant. The coagulant-coated hand mold was then dipped into the NR for 15 s and dried at room temperature for 2–3 min. The NR compounded latex film was then cured for 30 min at 120 °C, allowed to dry, and demolded. The toxicity of gloves containing mangosteen peel was lower than that of gloves containing silver nitrate, which can impact human skin and should be used in the appropriate ratio to prevent microbial infections. *E. coli*, *B. subtilis*, *S. aureus*, and *P. aeruginosa* were shown to be inhibited by mangosteen peel concentrations between 80 and 100 g/mL [93]. 

### 4.4. NR Films with Cellulose Nanocrystals as Reinforcing and Crosslinking Agent for Application in Gloves

**Figure 12:**
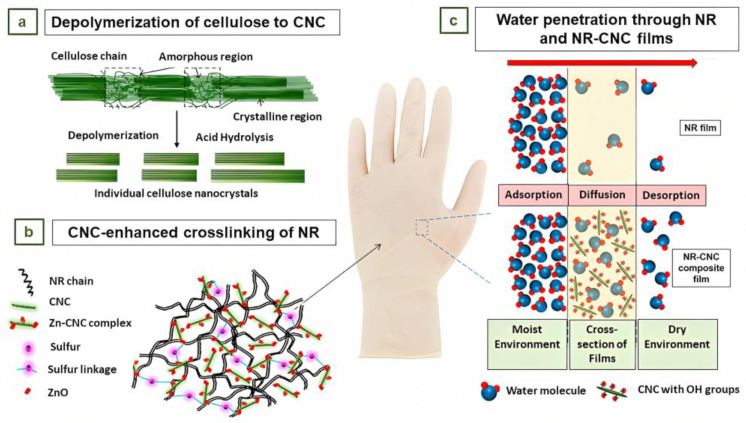
(**a**) Depolymerization of cellulose to nanocellulose (reprinted with permission under a Creative Commons license (CC BY 3.0) from reference [96]). (**b**) Illustration of the formation of a Zn–cellulose complex with CNC in the cross-linked NR matrix [99]. (**c**) Illustration of the proposed permeation mechanism through NR and NR–CNC nanocomposites and THF. (**b**,**c**) Reprinted and adapted with permission from reference [99]. Copyright © 2020 American Chemical Society.

Because of their elevated rigidity and reinforcing capacity, cellulose nanocrystals (CNCs) are a promising bio-filler. Typically, CNCs are obtained from renewable resources through acid hydrolysis, as is shown in Figure 12a [94,95,96]. CNCs are normally dispersed in NR latex without modification due to their great dispersibility in aqueous media, which is a result of their high content of hydroxyl groups [97]. However, it has been demonstrated that modifying the surface of CNCs enhances their reinforcement effect on NR. As a result of the hydrophobic–hydrophobic interaction between modified CNCs and NR, the tensile strength and the elongation at break increased significantly compared to unmodified CNCs. To ensure compatibility with the rubber while preserving the dispersion of CNCs aqueous media, it is crucial to strike a balance in the degree of modification of the CNCs [98]. 

Blanchard et al. studied the influence of CNCs on the reinforcing, crosslinking, and solvent barrier characteristics of lightly crosslinked NR films [99]. In nonpolar matrices, it is difficult to efficiently disperse CNCs due to their extensive surface area and their trend to form aggregates bonded together by hydrogen bonds. Therefore, for proper dispersion, it was necessary to prepare an aqueous colloidal suspension of CNCs [100]. 

As an initial step for experimentation, NR composite latex was prepared by predispersing the compound chemicals, including ZnO and sulfur, in water. This predispersion mixture was subsequently incorporated into NR formulations [99]. 

For NR-CNC films, CNCs were incorporated at concentrations of 0 (NR control), 0.5, 1.5, 3, and 5 phr. Dipping films were produced using glass substrates that were dipped in a coagulant solution for 10 s and then dried at 65 °C for 20 min. The substrate was then dipped for 40 s in the NR formulations and cured at 100 °C for 1 h. The cured films were then peeled off from the glass substrates and cured for an additional hour. The dynamic and tensile mechanical properties of these dipping films were analyzed. Increased crosslinking resulted in significant improvements in both tensile strength and modulus compared to the base NR control. The force required to break the films increased as film thickness decreased [99].

To prepare cast films, latex formulations containing 40 wt % total solids were cast on glass substrates to obtain dried NR films of 12 mm in thickness. The films were then cured at 100 °C for 1 h, peeled off, and post-cured for 1 h. The cast films were used to evaluate the impact of CNCs on morphology, crosslinking density, and barrier properties. The addition of CNCs resulted in an increase in the crosslinking density of the NR films. This was presumably attributed to increased dispersion of the crosslinking activator ZnO due to the development of a Zn–cellulose complex, with the CNCs acting as a dispersant (Figure 12b) [99].

The nanocomposite thin films had low permeability to nonpolar solvent vapors, such as tetrahydrofuran (THF), but high permeability to water vapor, as shown in Figure 12c. This ability of the material to reach or surpass NR strength at lower film thicknesses may allow for thinner gloves and for hand perspiration to pass through while functioning as a barrier to solvents. It may also lead to cost savings by reducing the use of NR. The findings of this investigation indicate that NR composite films produced using NR/CNCs have considerable potential for application as gloves [99].

### 4.5. NR and NBR Gloves Coated with Gardine Solution 

Gardine solution is an innovative antiseptic dye with broad-spectrum antibacterial effects prepared by combining brilliant green with chlorhexidine. Brilliant green and chlorhexidine, when used independently, have been shown to have low antimicrobial efficacy, but when combined, they have a synergistic effect with significantly improved efficacy. Chlorhexidine is a non-toxic chemical widely used in low concentrations in mouthwash solutions along with other antiseptics [4]. Historically, brilliant green has been used as a topical anti-infective for skin lesions and is currently used in combination with gentian violet and proflavine hemisulfate in neonatal nurseries as a broad-spectrum antiseptic solution [101]. 

**Figure 13:**
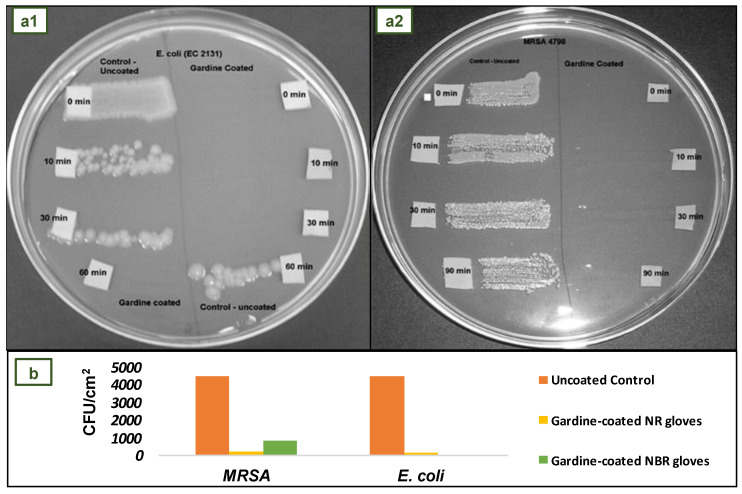
(**a1**) Brief exposure test of Gardine-coated gloves. (**a2**) Long-term exposure. (**b**) Mean colony counts recorded for all coated glove types after 24 h exposure to MRSA or *E. coli*. Reprinted and adapted with permission from reference [101]. Copyright © 2009 Elsevier.

In the study conducted by Reitzel et al., NR and NBR gloves were impregnated with Gardine solution to create antimicrobial coating. The results indicated that Gardine-coated NR and NBR gloves were highly effective in reducing pathogenic contamination in the short term and long term. For the short-term exposure test, 1 cm^2^ segments of NR and NBR coated and uncoated control gloves were exposed to 1.5 × 10^8^ colony-forming units (cfu)/mL of methicillin-resistant *Staphylococcus aureus* (MRSA), vancomycin-resistant enterococci, multidrug-resistant (MDR) *E. coli*, MDR *Acinetobacter baumannii*, and *Candida albicans*. The segments were dried for 30 s, 10 min, 30 min, and 1 h, and then distributed on agar plates, which were incubated overnight at 37 °C, and growth was measured. All microorganisms tested were significantly reduced within 30 s and completely eliminated within 1 h when exposed to Gardine-coated NR gloves. Figure 13(a1) shows the complete kill within 30 s for *E coli* and Figure 13(a2) for MRSA [101].

For the long-term exposure test, MRSA and *E coli* were employed because they are biofilm-forming microorganisms typically found in hospital environments. Figure 13b shows that the average number of MRSA and *E. coli* colonies adhered to the surface of Gardine-coated gloves was significantly lower than that of control gloves. After 24 h, the adhesion of MRSA and *E. coli* to the surface of Gardine-coated NR gloves decreased by at least 95%. On the surface of Gardine-coated NBR gloves, there was an 80% reduction in MRSA and a 100% reduction (total kill) in *E coli* [101]. 

These coated gloves represent an alternative means of preventing the spread of invasive microbial pathogens. In terms of final cost, the Gardine impregnation process would be carried out during the manufacture of the gloves, reducing the costs associated with a separate additional manufacturing process. In addition, Gardine solution is made up of low-cost components. These antimicrobial gloves would be cost-effective based on material and production time estimates [101].

### 4.6. NBR Gloves Coated with Poly(hexamethylene Biguanide) Hydrochloride

Poly(hexamethylene biguanide) hydrochloride (PHMB) is a positively charged polymer with antibacterial and antiviral activity [102]. It is effective against a wide range of pathogenic microorganisms, including Gram-negative bacteria, Gram-positive bacteria, and fungi [103]. Due to its strong and nonspecific interaction with negatively charged phospholipids in the cellular membranes of microorganisms, PHMB possesses a broad antibacterial spectrum [102]. PHMB has been utilized for decades with no reports of bacterial resistance [104]. It has been demonstrated to pose a minimal risk of skin sensitivity and a low toxicity risk to humans in general [102]. Moreover, PHMB has disinfectant and antiseptic properties [104], which makes it suitable for house cleaning, water sanitization, hygiene products, and wound treatment [103]. 

**Figure 14:**
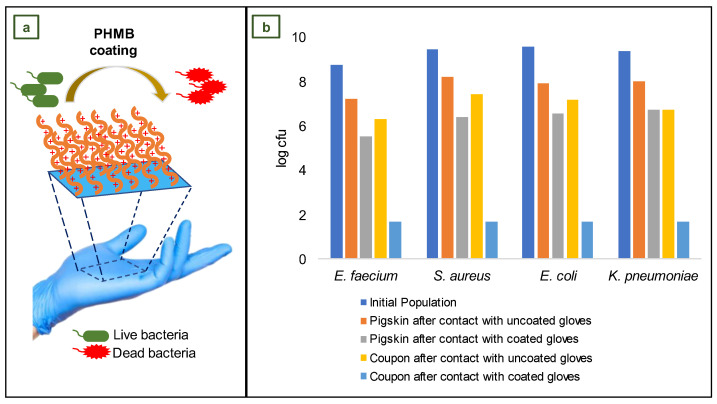
(**a**) Schematic illustration of coating (illustration prepared by the authors based on reference information [105]). (**b**) Pre- and post-exposure populations of challenge microorganisms following transfer procedures. Adapted with permission under a Creative Commons license (CC BY) from reference [105]. Copyright © 2013 Elsevier.

Leitgeb et al. conducted an in vitro examination of the antibacterial efficacy of a new non-sterile NBR medical glove coated with PHMB on its outer surface provided for Ansell Ltd. These gloves are intended for use during patient examinations to avoid microorganism cross-contamination across surfaces in healthcare environments. The study’s goal was to evaluate the performance of NBR medical gloves, with and without antibacterial PHMB coating on the outside surface, (Figure 14a) made from the same formulation [105]. 

For this investigation, the quantity of bacteria recovered from a stainless-steel coupon after touching a pigskin substrate with both gloves was evaluated. Pigskin substrates were contaminated with suspensions containing 1 × 10^9^ colony-forming units of *E. faecium* ATCC 51559, *E. coli* ATCC 25922, *K. pneumoniae* ATCC 4352, and *S. aureus* ATCC 33591. After impregnating sections of pigskin with bacterial suspensions, swatches of coated and uncoated (control) gloves were tightly pressed onto the inoculated pigskins. Immediately, a sterile weight was placed on the glove swatch and left in place for 1 min; then, the sample was placed in a sterile Petri plate with the exposed side facing up and left for 5 min at room temperature. The contaminated side of the glove swatch was then positioned on a sterile 40 mm diameter stainless steel coupon. The weight was immediately placed onto the test glove for 1 min. Separately, the contaminated pigskin, stainless steel coupon, and test glove swatch were placed in buffer solution and carefully vortexed [105]. 

Bacterial extractions were carried out on the pigskin substrate, stainless steel coupons, and each glove swatch, and the difference between the coated and uncoated control gloves was analyzed (Figure 14b). In comparison to the non-coated control glove, the coated glove reduced *E. faecium* recovery by 4.63 log cfu, *E. coli* recovery by 5.48 log cfu, *K. pneumoniae* recovery by 5.03 log cfu, and *S. aureus* recovery by 5.72 log cfu. According to these findings, the use of antibacterial medical gloves may be an innovative method for preventing or limiting cross-contamination and, consequently, the indirect spread of infections in intensive care unit (ICU) settings [105].

### 4.7. NR Antimicrobial Three-Layer Glove

In some instances, external coating is not suggested for surgical gloves since it might create undesired side effects. There is a possibility of transferring the coating to the patient’s tissues, cells, and organs during surgery. For these reasons, a three-layer glove is a good alternative for invasive procedures.

The three-layer antimicrobial coating method, used in surgical gloves, inserts antimicrobial chemicals between NR films. It is possible by triple-dipping the glove mold in NR compounded latex and antimicrobial solutions during the manufacturing process. Triclosan, nanocomposites, metal ion-based antimicrobial agents, vegetable oil surfactants, antiseptic dyes, chlorhexidine, gluconate, dodecyl dimethyl ammonium chloride salt, benzalkonium chloride, and similar antimicrobial agents might be incorporated in this manner [39].

**Figure 15:**
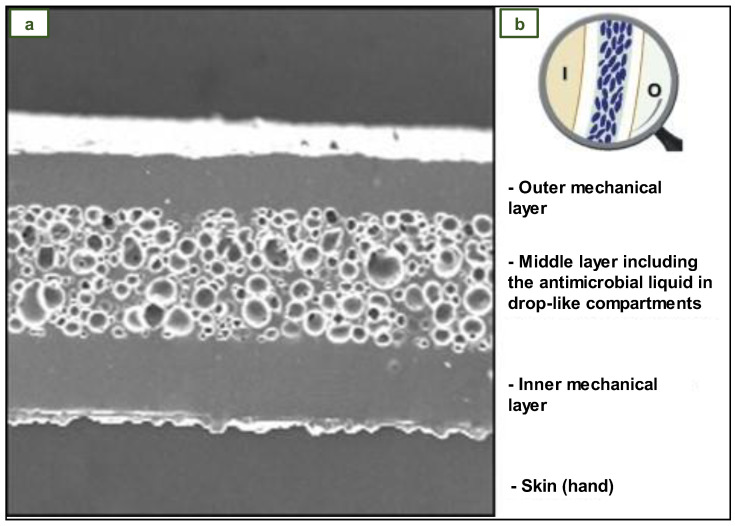
Three-layer NR glove with antimicrobial agent. (**a**) Cross-section micrograph. (**b**) Three-layer scheme. Reprinted with permission from reference [106]. Copyright © 2011 Elsevier.

Daeschlein et al. created a prototype of a new NR three-layer antibacterial surgical glove. Figure 15a shows a microscopic cross-sectional view of a droplet-like mixture of antimicrobial agents (chlorhexidine and quaternary ammonium salts) in the intermediate layer, while Figure 15b is a representation of the inner (I) and outer (O) surfaces adjacent to the rubber border layers. The antimicrobial agent is released from the interlayer upon penetration of the glove, resulting in deposition of the active antimicrobial agent at the site of damage or puncture. Because the antimicrobial agent droplets are trapped between two NR boundary layers, there is no continuous exposure of the material to the skin surface in the absence of lesions, hence lowering the possibility of sensitivity from extended contact [106].

### 4.8. NBR Antimicrobial Gloves Coated with Electrospun Trimethylated Chitosan (TMCh)-Loaded (PVA) Fibers

Usually, antimicrobial agents have been added to gloves through coatings. But after this treatment, the surface of the gloves tends to become smoother, and they tend to slip more when they are used. Thus, alternative coatings that make the surface of the glove rougher are needed. Ultrafine fibers, loaded with antibacterial agents, are one of the materials that solve this problem. Electrospinning is the method most often used to make these fibers because it provides the opportunity to conveniently control the fiber dimensions. This approach essentially utilizes an electric field to draw a polymer strand [107].

Chitosan is a highly biocompatible antibacterial agent composed of β-(1→4)-*D*-glucosamine and y β-(1→4)-*N*-acetyl *D*-glucosamine units. Water-soluble chitosan derivatives such quaternized chitosan (QCh) and alkylated chitosan like trimethylated chitosan (TMCh) are alternatives to chitosan alone (usually only soluble in acidic media) for use as antibacterial agents in neutral pH conditions [107]. The presence of lipoteichoic acids, a significant component of the cell wall of Gram-positive bacteria, and lipopolysaccharide, of the outer membrane of Gram-negative bacteria, which provide a linkage for polycationic TMCh and disrupt the membrane functions, may explain the antibacterial capabilities of TMCh [108]. Normally, lipopolysaccharide and proteins are kept together by electrostatic interactions with divalent cations, which are essential for the outer membrane stability. Polycations compete with divalent metals such as Mg^2+^ and Ca^2+^ ions in the cell wall, hence compromising the cell wall integrity [109]. 

Vongsetskul et al. effectively coated NBR gloves with ultrathin electrospun PVA fibers loaded with TMCh. Using water as a solvent, solutions containing 4% *w*/*v* of TMCh mixed with 8% *w*/*v* of PVA and 2% *w*/*v* of TMCh mixed with 10% *w*/*v* of PVA were prepared. These solutions were subjected to the electrospinning process using a feed rate of the solutions of approximately 0.5 mL/h [107]. 

Different electrical voltage values were used (12, 14, 16, 18, and 20 kV) to analyze its effect on the morphological appearance of the produced fibers. As the applied voltage increased from 12 to 16 kV, the fibers became smoother and smaller. SEM studies revealed that the optimal conditions to produce uniform fibers (101 to 133 nm of diameter) were a voltage of 16 kV and solution of 4% *w*/*v* TMCh-8% *w*/*v* PVA [107]. 

For the surface roughness and wettability study, film-coated NBR gloves were prepared by dipping in a 4% *w*/*v* TMCh-8% *w*/*v* PVA solution and drying at room temperature. The surface roughness was increased from 429 to 511 µm^2^ by coating electrospun fibers on the glove. The contact angle measurements of the NBR glove surface, TMCh-PVA film on the NBR glove surface, and TMCh-PVA electrospun fibers on the NBR glove surface were 80.1° ± 1.2°, 59.3° ± 8.9°, and 37.1° ± 2.7°, respectively. These values indicate that the hydrophilicity of the gloves increased when coated with TMCh-PVA films or TMCh-PVA fibers [107].

To evaluate the antimicrobial activity of the fiber-coated gloves, the agar plate method was used. *E. coli*, *P. aeruginosa*, *A. baumannii*, and *C. albicans* were tested. In the results of antimicrobial testing, a zone of growth inhibition against the tested microbes by the TMCh-PVA fiber-coated NBR gloves was observed, whereas no antimicrobial activity was observed for the PVA fiber-coated ones. In conclusion, NBR gloves coated with these TMCh)-loaded (PVA) fibers exhibited antibacterial properties against Gram-negative bacteria, including *E. coli*, *P. aeruginosa*, and *A. baumannii*, as well as yeast *Candida albicans*. Likewise, this coating on the external surface of the glove improved roughness and wettability, which would be advantageous for gripping and practical applications [107].

### 4.9. Antibacterial NR Films with Surface-Anchored QP4-VP for Application in Medical Gloves

**Figure 16:**
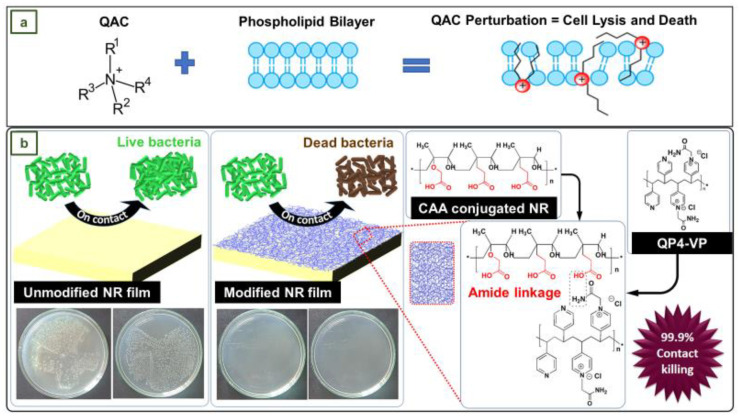
(**a**) Mode of action of QACs against both bacterial and viral phospholipid membranes (Reprinted with permission under standard ACS Author Choice/Editors’ Choice usage agreement from reference [110]). (**b**) Antibacterial activity of QP-4VP-conjugated NR films vs. Control NR films. Reprinted with permission from reference [111]. Copyright © 2022 Elsevier.

Quaternary ammonium compounds (QACs) are cationic active biocides, which, in addition to their antibacterial action, are ideal for cleaning and deodorizing [4]. The mechanism of action of QACs against bacterial and viral phospholipid membranes is depicted in Figure 16a, where the red spheres represent positively charged nitrogen atoms. When bacteria encounter cationic ammonium agents, several processes take place: first, QACs connect to and insert themselves into the cell wall; then, they interact with the cytoplasmic membrane, releasing cytoplasmic material outside the membrane; and finally, they cause the cell wall to disintegrate via autolytic enzymes. In general, the loss and destruction of various sections of the bacteria results in their inactivation [4,110].

In the work of Arakkal et al., NR films were converted into an effective antibacterial material (Figure 16b) through surface conjugation of quaternized poly(4-vinylpyridine) (QP4-VP) via an amide linkage bond using chloroacetic acid. The antibacterial action of poly(4-vinylpyridine) has been extensively examined and explored in ion exchange resins, but its low biocompatibility prevents its widespread application in biomedicine. However, it has also been shown that with the right choice of space groups and copolymerization, the hemolytic activities of the polyelectrolyte can be inhibited while maintaining antibacterial activity [111].

To evaluate the antimicrobial activity and stability of the NR films coated with a QP4-VP-conjugated surface layer, they were subjected to a leaching process in milli-Q water at 50 °C for 4 days. Subsequently, coated NR and leached coated NR films were exposed to *P. aeruginosa* and *A. baumannii* strains [111]. 

The results indicated that the microbial load of *P. aeruginosa* was reduced by 93.25% and 99.98% with the coated NR films and leached coated NR films, respectively. Similarly, the reduction in *A. baumannii* was 32.41% and 99.99%. The improved bacterial reduction rate confirmed that the leaching process at elevated temperatures allows the disoriented QP4-VP chains to organize efficiently, resulting in a higher conjugation density. This conjugation method could be used to develop similar antibacterial surfaces for various applications, such as medical gloves [111]. 

### 4.10. NR, NBR, and PE Medical Gloves with Blood-Repellent, Antibacterial, and Wound Healing Properties, Modified through Spraying Process 

Medical blood-repellent gloves (MBRGs) were proposed by Zhuo et al., by means of treating the surface of conventional NR, NBR, and PE medical gloves with a novel procedure to achieve blood repellency and promote wound healing. This treatment was executed with a mist spray (MS), which was elaborated by mixing sodium citrate (SC), didecyldimethylammonium chloride (DDAC), and a silicon oil emulsion (SOE) containing aminoethylaminopropyl polydimethylsiloxane (AEAPS). It was intended that SC would combine with blood calcium ions to inhibit blood coagulation and glove adhesion, that AEAPS would be responsible for the hemophobicity and hydrophobicity of the treated gloves, and that DDAC, being a quaternary ammonium compound, would endow the gloves with antibacterial properties [112]. 

MBRGs were created by spraying MS onto the surface of commercial NR, NBR, or PE medical gloves and waiting for one minute. Experiments in vitro and in vivo demonstrated that these gloves are hemophobic and facilitate the healing of infected wounds. The antibacterial efficiency of MBRGs was tested against known bacteria strains. In vitro antimicrobial testing was performed with MS concentrations of 800, 400, 200, 100, and 50 g/mL. A solution of *S. aureus* or *E. coli* was added to each MS concentration and incubated first in tubes and then on agar plates. Phosphate-buffered saline (PBS) was used instead of MS in the control group. After 24 h, the antibacterial efficacy was assessed. MS showed outstanding activity against *S. aureus*, with an antibacterial rate close to 100% at a concentration of 50 μg/mL. In the case of *E. coli*, the antibacterial effect was close to 100% when the concentration was 200 μg/mL. The antibacterial activity of MS was also verified through the live/dead viability assay. In this study, *S. aureus* and *E. coli* were treated with MS. After treatment with MS, red fluorescence (dead bacteria) was clearly visible, whereas blue fluorescence (living bacteria) was nearly non-existent [112].

### 4.11. NR Gloves with SiO_2_ and ZnO Hybrid Nanofillers 

Silicon dioxide (SiO_2_) or silica is a well-known reinforcing filler in the rubber industry and is commonly used to improve the physical and mechanical properties of NR [113]. To achieve the desired result of reinforcement, conventional silica fillers must be applied in high quantities. Studies have shown, however, that when the size of SiO_2_ particles reaches the nanometer range, the nanoparticles can not only drastically reduce the filler content, but also provide superior reinforcement effects [114]. 

Zinc oxide (ZnO) is an n-type semiconducting particle with catalytic, electrical, and optical properties. This material has a broad UV absorption spectrum, good photostability, thermal stability, and biocompatibility. Due to the nano-size effect, ZnO has photocatalytic antibacterial properties when its size reaches the nanoscale. When ZnO is exposed to UV light, the photon energy is higher than the bandgap energy, which causes the valence band electrons to gain energy and migrate. As a result, many electron–hole pairs are generated on the nano-ZnO surface [115]. 

The holes (h^+^) created on the surface of nano-ZnO generate reactive oxygen species (ROS) when they combine with water or oxygen from the air. These oxidative species adsorb onto the surface of the nanoparticles. Some studies have determined that the interaction between ROS and cells is the key antibacterial mechanism of nano-ZnO [115]. There are indications that antibacterial activity can be initiated not only by UV rays, but also by ambient light [116]. Furthermore, when microorganisms come into contact with nano-ZnO, the released Zn^2+^ and the sharp edges of ZnO nanoparticles can rupture their cell walls [117].

Mou et al. investigated the combination of the exceptional functional capabilities of nano-SiO_2_ and nano-ZnO as fillers of NR to create medical gloves. The ZnO and SiO_2_ nanoparticles used had an average particle size of about 78 nm and 65 nm, respectively. To evenly distribute the fillers in a composite nano-dispersion, the researchers created a high-speed and high-pressure nano-disperser. To obtain experimental glove samples using the dipping method, NR latex, composite nano-dispersion, and compounding chemicals were thoroughly combined. Initially, the cleaned and dried mold was dipped for 5 s into the previous mixture, then dried at 85 °C for 20 min, and then leached in water at 75 °C for 30 s. After hemming, the gloves were dried at 120 °C for 40 min. Finally, after demolding, they were placed in a drum drier and vulcanized at 120 °C for 20 min [114]. 

**Figure 17:**
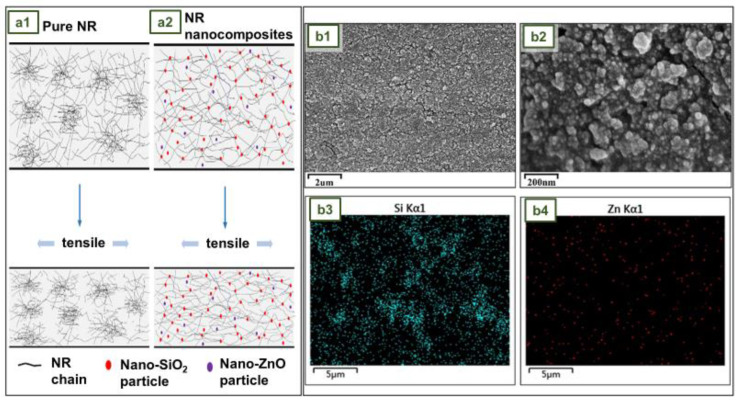
(**a1**,**a2**) Schematic diagram of nanoparticle enhancement mechanism. Scanning electron microscopy images of (**b1**,**b2**) LZ1S4.2 and element distribution of (**b3**) Si and (**b4**) Zn. Reprinted with permission from reference [114]. Copyright © 2022 John Wiley and Sons.

The results indicated that the uniform dispersion of nano-SiO_2_ filler enhanced the amount of molecular chain entanglements in the NR, as depicted in Figure 17(a1,a2), making the material structure more compact and improving the barrier performance and aging resistance. The combination NR fillers with 1 phr of ZnO and 4.2 phr of SiO_2_ reported the highest tensile strength of 32.6 MPa and elongation at break of 957% in mechanical properties tests. These results represented an interesting improvement in tensile strength and elongation at break compared to the unfilled NR sample, whose values were 27.8 MPa and 880%, respectively. Figure 17(b1,b2) shows scanning electron microscopy images at different magnifications. In addition, the elemental distribution of the LZ1S4.2 sample is presented in Figure 17(b3,b4) [114].

Strains of *E. coli* and *S. aureus* were chosen for antibacterial testing. The findings of the antibacterial activity of NR gloves are described in Table 5. The quantity of bacteria on the blank immediately after inoculation is given by *U*_0_, whereas the quantity of bacteria on the blank and on the antibacterial sample after 24 h of incubation are *U_t_* and *At*, respectively. The antibacterial activity *R* is equivalent to *log U_t_ - log A_t_*. A value of *R* greater than 2 shows that the antimicrobial test is passed. After 24 h of culture, nano-ZnO-treated samples had virtually no bacteria. The *R* values for *E. coli* and *S. aureus* were more than 99.9% and greater than 5.2, respectively. Furthermore, the NR gloves containing hybrid nanofillers remained biocompatible. Therefore, this NR/ZnO/SiO_2_ nanocomposite may have applications in the development of other NR products [114].

### 4.12. NR Antimicrobial Gloves Impregnated with Biosynthesized Silver Nanoparticles 

Ionic silver (Ag^+^) has long been recognized as an antibacterial metallic element capable of acting against bacteria such as *E. coli* [4,118]. To take advantage of silver ion activity, silver nitrate (AgNO_3_), a solid powder with antiseptic qualities, is frequently utilized. It can be applied as a surface coating on various items to eliminate viral and bacterial cells [93]. The specific mechanism of silver antibacterial activity has not yet been fully understood. Among the variety of approaches, the main three of several pathways that determine the antibacterial activity of silver nanoparticles are the following: (1) irreversible bacterial cell membrane damage caused by direct contact; (2) production of reactive oxygen species (ROS); and (3) interaction with DNA and proteins [119]. Nanoparticle size plays an important role in antibacterial activity. It has been shown that the smaller the size of the nanoparticle, the greater its ability to penetrate bacteria. The nanoparticles attach to the bacterial cell wall, penetrate it, and cause damage and alterations in various metabolic pathways. It affects DNA replication and protein synthesis; due to oxidative stress, ROS generation occurs, which eventually leads to cell death [120].

Paosen et al. developed NR gloves coated with biosynthesized silver nanoparticles (AgNPs). The biosynthesis of AgNPs was performed using extract of *Eucalyptus citriodora* ethanolic leaf. NR gloves were cut into pieces, dipped into the AgNP solution, and dried. The elemental analysis of the coated gloves revealed that 24.8% wt silver was firmly adhered to the surface. Biofilms of *S. aureus* ATCC 25923, *P. aeruginosa* ATCC 27853, and *Candida albicans* ATCC 90028 were expected to develop on glove samples during 24 h of incubation [121]. 

**Figure 18:**
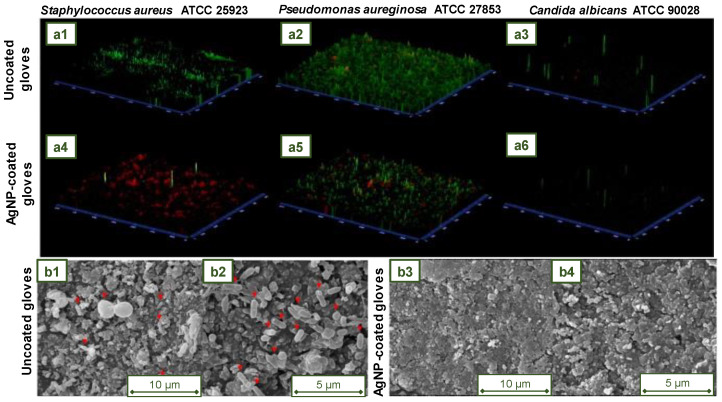
Fluorescence microscopy images of (**a1**,**a4**) *S. aureus* ATCC 25923, (**a2**,**a5**) *P. aeruginosa* ATCC 27853, and (**a3**,**a6**) *C. albicans* ATCC 90028 biofilms incubated with (**a1**–**a3**) uncoated gloves and (**a4**–**a6**) AgNP-coated gloves. Scanning electron microscopy (SEM) analysis. SEM micrograph of polymicrobial anti biofilm activity of (**b1**,**b2**) uncoated gloves and (**b3**,**b4**) AgNP-coated gloves. Reprinted with permission from reference [121]. Copyright © 2021 John Wiley and Sons.

Figure 18(a1–a6) shows the differences in the effect of staining microbial biofilms with uncoated gloves and AgNP-coated gloves. The results revealed that AgNP-coated gloves effectively removed *S. aureus* biofilms. Images from fluorescence microscopy were stained with a red fluorescent DNA-specific dye that only penetrates cells with damaged membranes and dead bacteria. The pictures revealed that *P. aeruginosa* cells were significantly less abundant and viable when cultured on AgNP-coated gloves compared to uncoated gloves, suggesting that AgNPs killed and inhibited microbial adhesion [121].

Figure 18(b1–b4) shows the scanning electron micrographs taken to examine the surface morphology of the bacterial adhesion by the mixed culture of *P. aeruginosa*, *S. aureus*, and *C. albicans* after 24 h of incubation. At magnifications of 5000 and 10,000, microbial cells on glove surfaces (indicated by red arrows) could be observed. Polymicrobial cells were fixed and colonized in the untreated gloves, whereas microbial cells were observed in tiny quantities on the coated surfaces. The viability of gloves coated with AgNPs was proven. These gloves presented significant antimicrobial activity, particularly against multidrug-resistant bacteria, and may be suitable for preventing or minimizing cross-contamination and indirect pathogen transmission in hospital settings [121].

### 4.13. NR Antimicrobial Gloves with Poly(dimethylsiloxane)-Copper Coating

Multiple studies have demonstrated that copper (Cu) possesses antimicrobial properties against *E. coli*, *L. monocytogenes*, *C. difficile*, yeasts, and viruses [122]. Cu-containing surfaces have been found to reduce environmental microbial contamination [123]. Cu has also been demonstrated to be a powerful antibacterial agent that can inhibit the growth of antibiotic-resistant bacteria such as MRSA, EMRSA-1, and EMRSA-16 [124,125].

In the research of Tripathy et al., an antimicrobial coating consisting of poly(dimethylsiloxane) (PDMS) combined with copper hydroxide nanowires (PDMS Cu) was developed. PDMS-Cu’s antibacterial properties have been demonstrated to reduce the viability of a panel of multidrug-resistant clinical pathogens (*E. coli*, *S. aureus* (MRSA), and *K. pneumoniae*). The PDMS-Cu surface exhibited superior activity as an antimicrobial film compared to the control (a glass coverslip). The antibacterial effectiveness of the PDMS-Cu surface was also evaluated in a patient room alongside controls (glass coverslips and PDMS substrates). On this occasion, PDMS-Cu was found to have the lowest amount of attached bacterial colonies compared to the controls [126]. 

In addition to the previous tests, it was established that coating a stethoscope diaphragm with a thin layer of PDMS-Cu could inhibit the transfer of infections from one patient to another in a hospital setting. The possibility of coating commercially available NR gloves with a thin layer of PDMS-Cu provides compelling evidence and an attractive opportunity to introduce antibacterial gloves into hospitals to minimize the transmission of nosocomial infections [126].

**Figure 19:**
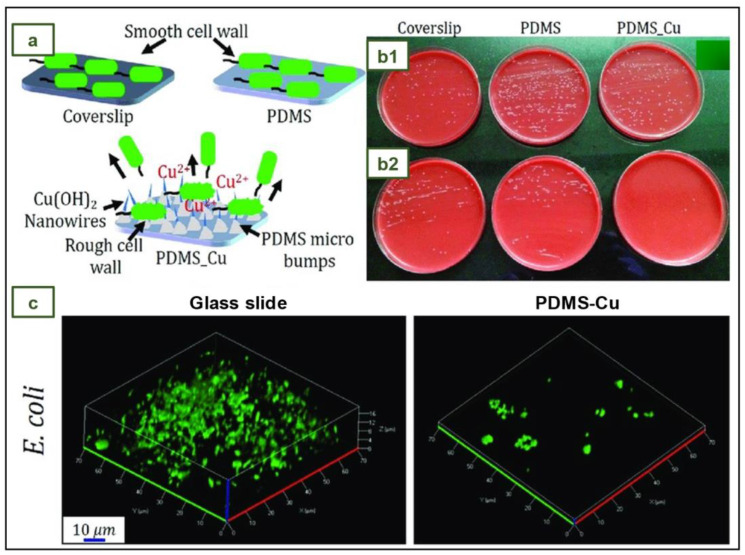
Images of the antibacterial activity of PDMS-Cu. Reprinted with permission from reference [126]. Copyright © 2018 American Chemical Society.

Figure 19a presents a schematic explanation of the antibacterial behavior of the PDMS-Cu surface. Figure 19(b1,b2) shows bacterial colonies on chocolate agar plates after 2 and 4 h (respectively) of exposure to the environment in a patient room. Figure 19c shows the confocal microscopy of *E. coli* biofilm on coverslip and PDMS-Cu surfaces after 5 days of incubation in Luria broth (LB) culture media [126]. 

## 5. Conclusions 

The sustainability of medical glove production processes is essential to support the three pillars of sustainability in the environmental, social, and financial sectors. In this sense, the use of performance-enhancing materials such as biomaterials, bio-fillers, biodegradable polymers, antimicrobial agents, etc. becomes a promising route to create new medical gloves with improved properties.

The integration of antiseptic substances or drugs with antimicrobial properties represent a viable option against drug-resistant bacteria and cross-contamination of pathogenic microorganisms and viruses. In this regard, ascorbic acid, biguanides, quaternary ammonium compounds, chitosan derivates, chlorhexidine, and Gardine solution have been successfully assayed. Furthermore, nanoparticles of metals, especially silver and copper, as well as metallic oxides such as ZnO and CuO have been effectively employed.

The use of low-cost bio-fillers such as sago starch, mangosteen peel, and cellulose nanocrystals can improve the biodegradability properties of gloves without sacrificing the softness, film thickness, and mechanical characteristics.

When scaling the knowledge obtained in the laboratory to industry, it is important to consider the ease of production and the profitability of the modifications. In this regard, processes that incorporate performance-enhancing materials directly into rubber formulations or that only require an extra dipping process may be the most attractive from an economic point of view. In summary, the optimization of crucial manufacturing parameters is necessary to obtain safe and high-quality gloves that meet regulatory criteria and are attractive to consumers and investors.

## Figures and Tables

**Table 1:** Typical chemicals used for latex stabilization [27].

Function	Description
pH increasing	Generally, KOH is added to latex to raise its pH to 10–11.
Surfactants	Suspensions of chemicals in water can be made more stable with the help of ionic and non-ionic additives.
Rosin resins	Some synthetic latexes, such as CR and IR, are formulated with colophonium resins, which effectively perform the functions of particle stability and film forming.

**Table 2:** Properties of main medical gloves.

Material	Advantages	Disadvantages
NR	Good resistance to alkali and acids. Comfortable, good fitting and feeling for hands. High elasticity and ability to adapt to shapes. High tear strength. Waterproof.	Permeable to several solvents. Poor resistance to chemicals. Possible allergies due to residual protein.
IR	Absence of allergy associated with proteins in NR gloves. Good elasticity and break resistance.	It is costly.
NBR	Good alternative for people that are allergic to NR gloves. Resistance to various chemicals, especially oils, fuels, weak acids, caustics, and some organic solvents. Eligible for handling most food materials. Good resistance to mechanical stress.	It has a low level of sensitivity, which may restrict how well the hands adapt to and operate with the gloves. Low resistance to alcohols, amines, ketones, ester, ethers, concentrated acids, halogenated hydrocarbons, and aromatic hydrocarbons.
CR	Resistance to temperature and harsh chemicals. Mechanical and flammability resistance are superior to NBR gloves. CR gloves fit and feel like NR gloves. Appropriate for people allergic to NR.	It is costly.
PE	Can be used for food material. Inexpensive option.	Poor resistance and barrier protection.
PVC	It is cost-effective, since PVC is inexpensive. Good for those suffering from skin and chemical allergies as it is skin-friendly.	Due to plasticizer, not adequate for handling fatty food since there is the possibility of migration of the plasticizer into the food. Less stretch, comfort, and elongation than NR. Poor resistance to chemical degradation. High permeability to chemotherapy drugs.

**Table 3:** Mechanical properties of examination medical gloves according to ASTM standards.

Property of Examination Gloves	ASTM D3578—19 (NR) [68]				ASTM D6319—19 (NBR) [69]		ASTM D6977—19 (CR) [70]	
	Before Aging		After Aging		Before Aging	After Aging	Before Aging	After Aging
	Type I	Type II	Type I	Type II				
Minimum Tensile Strength (MPa)	18	14	14	14	14	14	14	14
Maximum Stress at 500% Elongation (MPa)	5.5	2.8	-	-	-	-	-	-
Minimum Ultimate Elongation (%)	650	650	500	500	500	400	500	400

**Table 4:** Examples of mechanical properties of KOSSAN medical gloves [72].

Property	Latex Examination Glove PS60Y		Nitrile Examination Glove CS30	
	Unaged	Aged	Unaged	Aged
Tensile Strength (MPa)	20–24	16–20	28–32	29–33
Ultimate Elongation (%)	700–740	600–640	500–540	460–500
Force at Break (N)	7.0–7.5	7.0–7.5	6.0–6.3	6.0–6.3

**Table 5:** Antibacterial test results of gloves [114].

Microorganisms Tested	*U*_0_ (cfu/cm^2^)	*U_t_* (cfu/cm^2^)	*A_t_* (cfu/cm^2^)	R	Antibacterial Rate (%)
*E. coli*	2.1 × 10^4^	2.9 × 10^5^	<0.6	>5.3	>99.9
*S. aureus*	2.1 × 10^4^	2.3 × 10^5^	<0.6	>5.2	>99.9
